# Metabolite Shifts Induced by Marathon Race Competition Differ between Athletes Based on Level of Fitness and Performance: A Substudy of the Enzy-MagIC Study

**DOI:** 10.3390/metabo10030087

**Published:** 2020-03-01

**Authors:** Jana F. Schader, Mark Haid, Alexander Cecil, Julia Schoenfeld, Martin Halle, Arne Pfeufer, Cornelia Prehn, Jerzy Adamski, David C. Nieman, Johannes Scherr

**Affiliations:** 1 School of Medicine, Department of Prevention, Rehabilitation and Sports Medicine, Technical University of Munich, 80992 Munich, Germany; Julia.Schoenfeld2@mri.tum.de (J.S.); Martin.Halle@mri.tum.de (M.H.); Johannes.Scherr@balgrist.ch (J.S.); 2 Department of Biomedical Development, AO Research Institute Davos, 7270 Davos Platz, Switzerland; 3 Research Unit Molecular Endocrinology and Metabolism, Helmholtz Zentrum München, Ingolstädter Landstraße 1, 85764 Neuherberg, Germany; mark.haid@helmholtz-muenchen.de (M.H.); alexander.cecil@helmholtz-muenchen.de (A.C.); prehn@helmholtz-muenchen.de (C.P.); adamski@helmholtz-muenchen.de (J.A.); 4 DZHK (German Centre for Cardiovascular Research), Partner site Munich Heart Alliance, Biedersteiner Str. 29, 80802 Munich, Germany; 5 Institute of Bioinformatics and Systems Biology, Helmholtz Zentrum München, German Research Center for Environmental Health, Ingolstädter Landstraße 1, 85764 Neuherberg, Germany; arne.pfeufer@web.de; 6 MVZ Molekulardiagnostik (Center for Molecular Diagnostics), 81543 Munich, Germany; 7 Lehrstuhl für Experimentelle Genetik, Technische Universität München, 85350 Freising-Weihenstephan, Germany; 8 Department of Biochemistry, Yong Loo Lin School of Medicine, National University of Singapore, 8 Medical Drive, Singapore 117597, Singapore; 9 North Carolina Research Campus, Appalachian State University, Kannapolis, NC 28081, USA; niemandc@appstate.edu; 10 University Center for Prevention and Sports Medicine, University Hospital Balgrist, University of Zurich, 8008 Zurich, Switzerland

**Keywords:** metabolism, biomarker, exercise, amino acids, fatty acids, urea cycle

## Abstract

This study compared metabolite shifts induced by training for, participation in, and recovery from a marathon race competition among athletes divided into three groups based on fitness (relative maximum oxygen uptake (VO_2_max)) and performance levels (net running time). Plasma samples from 76 male runners participating in the Munich Marathon were analyzed for metabolite shifts using a targeted metabolomics panel. For the entire cohort of runners, pronounced increases were measured immediately after the race for plasma concentrations of acylcarnitines (AC), the ratio (palmitoylcarnitine + stearoylcarnitine)/free carnitine that is used as a proxy for the activity of the mitochondrial enzyme carnitine palmitoyltransferase, and arginine-related metabolites, with decreases in most amino acids (AA) and phospholipids. Plasma levels of AA and phospholipids were strongly increased 24 and 72 h post-race. Post-race plasma concentrations of AC and arginine-related metabolites were higher in the low compared to top performers, indicating an accumulation of fatty acids and a reliance on protein catabolism to provide energy after the marathon event. This study showed that marathon race competition is associated with an extensive and prolonged perturbation in plasma metabolite concentrations with a strong AC signature that is greater in the slower, less aerobically fit runners. Furthermore, changes in the arginine-related metabolites were observed.

## 1. Introduction

Interest in extreme sports events like (ultra-)marathons, ultratrails, and triathlons has risen, and includes participants who vary widely in performance capabilities and training volumes and regimens [1,2,3]. Advances in mass spectrometry (MS) during the past decade have allowed investigators to use targeted- and non-targeted metabolomics to better understand the metabolic response to intense and prolonged exercise [4,5,6,7,8,9]. These studies have shown that intense exercise of two hours duration and longer causes an extensive shift in hundreds of metabolites, especially those from the lipid biochemical pathway. Most of these studies, however, have been laboratory-based with relatively small numbers of study participants and recovery blood samples, and few have compared responses based on race performance or fitness level. In one of the earliest studies, Lewis et al. used a targeted metabolomics approach to analyze 25 runners who participated in the Boston Marathon [10]. They observed a reduction in glucogenic amino acids and an increase of metabolites related to lipolysis and ketone body production, and these findings have been confirmed in more recent studies using improved MS technology [6,7,8,11,12]. Post-race concentrations of plasma glycerol were highest among runners with a higher relative maximum oxygen uptake (VO_2_max) compared to those with lower aerobic capacities. These data supported higher levels of lipolysis in higher trained athletes following strenuous exercise.

We sought to extend the findings of Lewis et al. [10] by using a targeted-metabolomics approach to measure metabolite shifts in 76 male runners five weeks before and for three days after competing in the Munich Marathon. The runners were divided into three groups based on aerobic fitness and race performance, and metabolite shifts were compared between groups. The targeted metabolomics panel was focused on quantification of 188 metabolites including acylcarnitines, amino acids, biogenic amines, hexoses, glycerophospholipids, and sphingolipids.

## 2. Results

### 2.1. Characteristics of Study Participants

**Figure 1:**
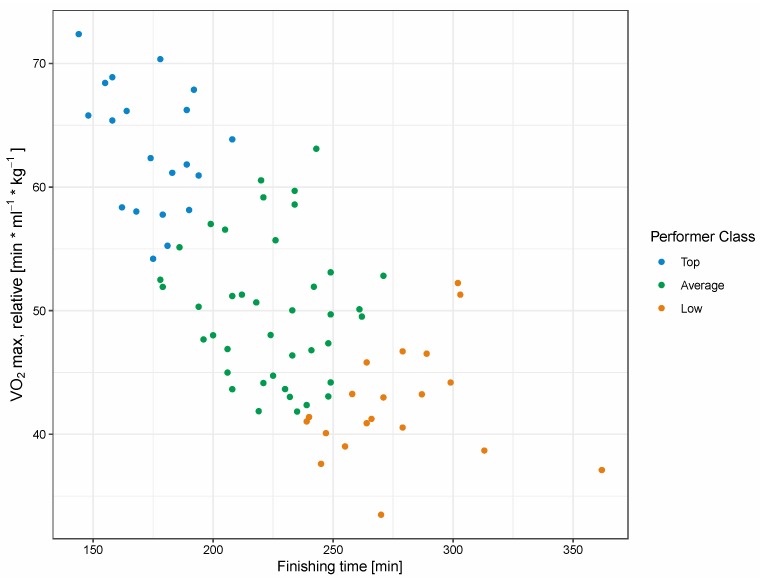
Performer selection plot. Stratification of the cohort based on the quotient between relative VO_2_max and running time. Athletes were categorized into 20 top (blue), 40 average (green), and 20 low (red) performers.

Participants were classified into three groups based on their peak aerobic capacity (VO_2_max) and finishing time of the marathon race (Figure 1). Table 1 shows the anthropometric and performance characteristics of the cohort after stratification. The top performers (TP) group was on average approximately six and 17 years younger than the average performers (AP) and low performers (LP) groups, respectively. VO_2_max ranged from 42 (LP) to 63 (TP) mL × min^−1^ × kg^−1^ and varied by approximately 10 mL × min^−1^ × kg^−1^ between the performance groups. The groups also differed significantly in body mass index (BMI), body fat [13] and marathon race time.

### 2.2. General Concentration Trends at Different Periods of Exercise

**Figure 2:**
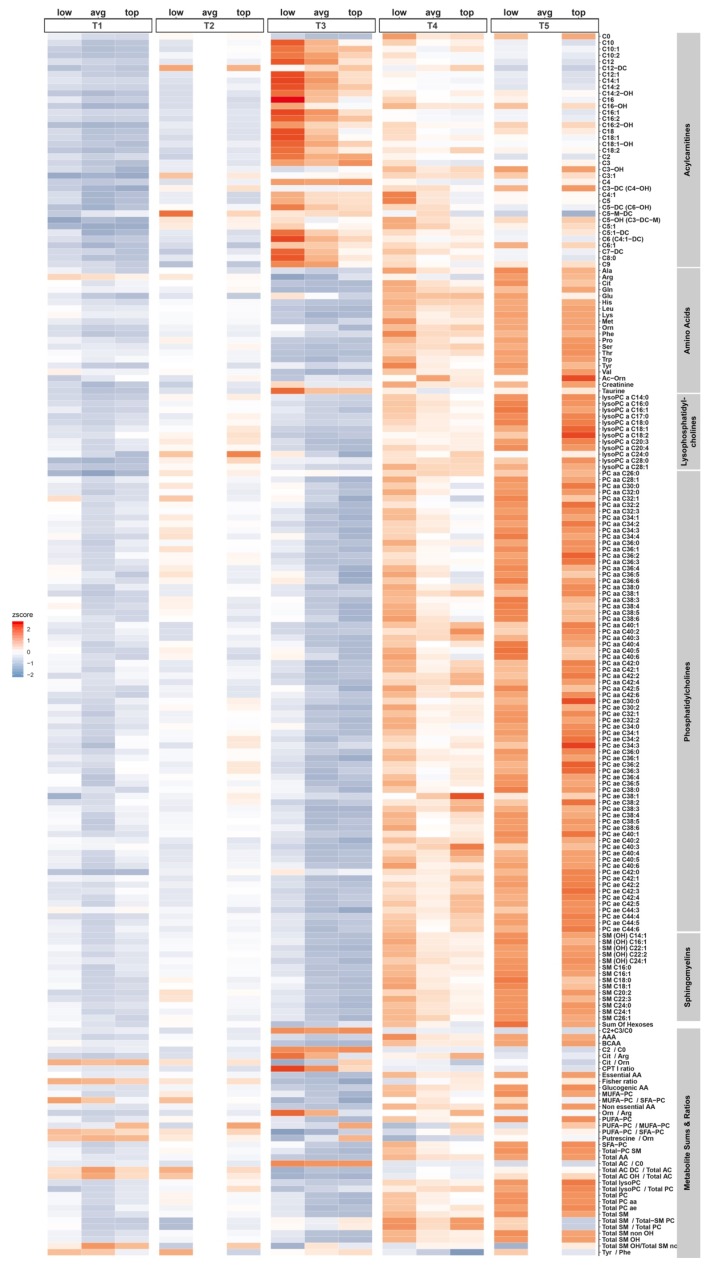
Heatmap of metabolite-wise auto-scaled (z-transformed) metabolite concentrations. Quantification of 188 metabolites including acylcarnitines, amino acids, biogenic amines, hexoses, phospholipids (lysophosphatidylcholines, phosphatidylcholines), sphingolipids (sphingomyelins), and metabolite sums and ratios. Groups included top (*n* = 20, top performers, TP), average (*n* = 40, average performers, AP), and low (*n* = 20, low performers, LP) performers based on endurance capacity (relative VO_2_max) and net marathon finishing time. Blood samples were collected at time points T1 (training phase), T2 (tapering phase), T3 (immediately after the race), T4 (24 h post-race), and T5 (72 h post-race). All blood samples were measured for the TP and LP groups, with T1, T3, and T4 measured for the AP group.

Plasma metabolite concentrations were auto-scaled across time points (Figure 2), with performance group specific log2-fold changes referenced to time point 1 (Figure S2, Table S1). While changes between time points 1 (T1, training phase) and 2 (T2, tapering phase) were subtle, a significant increase of almost all acylcarnitines and a related ratio ((C18 + C16)/C0) (palmitoylcarnitine + stearoylcarnitine)/free carnitine) that is used as proxy for the activity of the mitochondrial enzyme carnitine palmitoyltransferase (CPT1) was observed immediately after the race (T3). Furthermore, the ratios of (C2 + C3)/C0 ((acetylcarnitine + propionylcarnitine)/free carnitine) and C2/C0, which reflect overall and even-numbered fatty acids beta-oxidation, respectively, were significantly increased (Figure S2, Table S1). During the recovery and compensation phases (T4, T5) acylcarnitines steadily decreased towards pre-race levels. 

**Figure 3:**
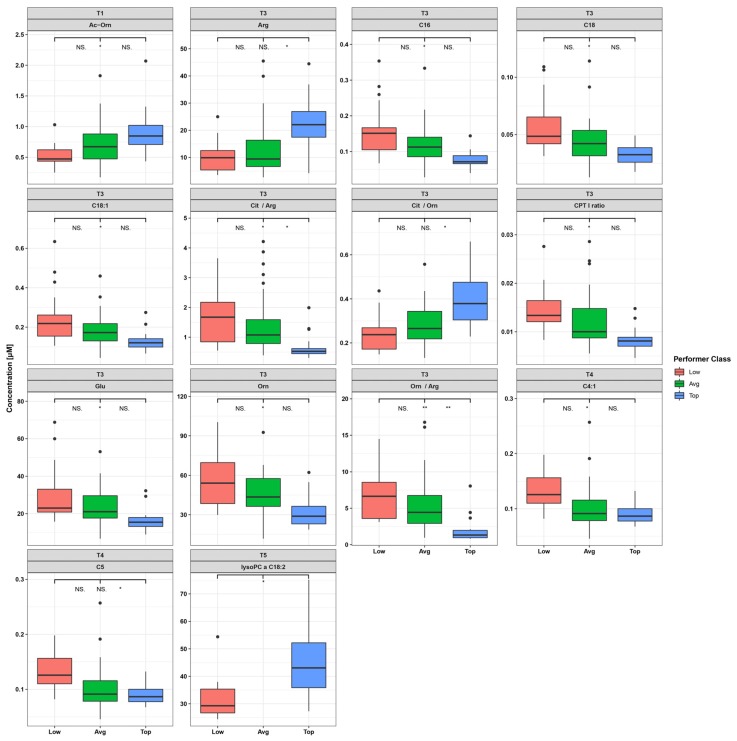
Significant metabolites (*q* < 0.05) after comparison of top, average, and low performers per time point. T1 = time point 1 (5 weeks before the race), T3 = time point 3 (immediately after the race), T5 = time point 5 (72 h after the race). The asterisks refer to pairwise comparisons with a Wilcoxon test procedure after Benjamini–Hochberg correction. Asterisks on the left side denote a significant difference between low and average performers, asterisks in the middle denote a significant difference between low and top performers, and asterisks on the right side denote a significant difference between average and top performers. * *q* < 0.05, ** *q* < 0.01, *** *q* < 0.001, NS. Non-significant.

**Figure 4:**
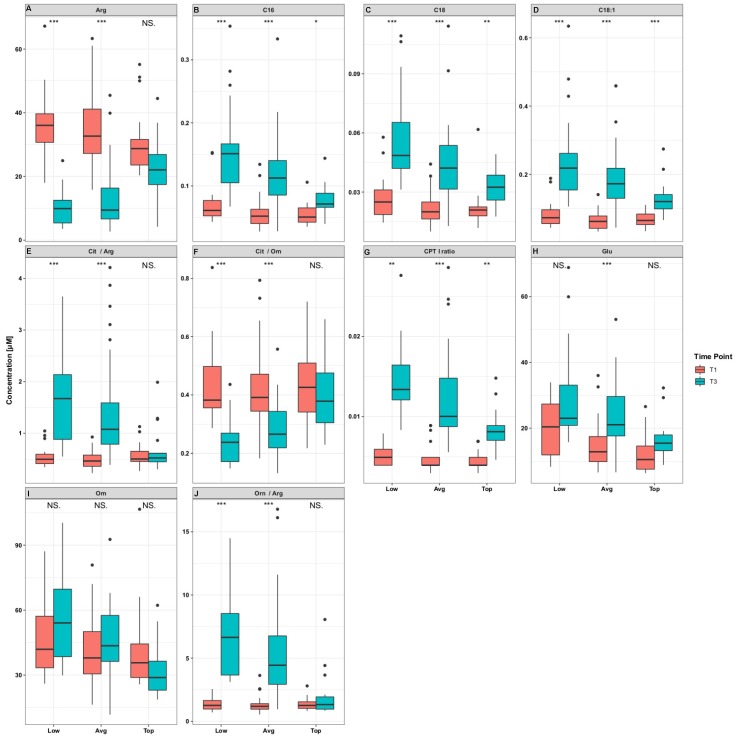
Significant metabolites (*q* < 0.05) after comparison of top, average, and low performers at time points T1 and T3. T1 = 72 h before the race (training phase), T3 = immediately after the race. In most instances, the differences between T1 and T3 were less pronounced in top performers in comparison to average and low performers. The asterisks refer to pairwise comparisons with a Wilcoxon test procedure after Benjamini–Hochberg correction. Asterisks denote a significant difference between time point 1 and time point 3 for each individual performer class. * *q* < 0.05, ** *q* < 0.01, *** *q* < 0.001, NS. Non-significant.

Plasma amino acid concentrations were generally decreased immediately post-race, with the exceptions of glutamate and some urea-cycle related metabolites such as ornithine and related ratios (e.g., ornithine/arginine ratio) (Figure 2, Figure 3 and Figure 4, Table S1). During the first 72 h of recovery, plasma concentrations of nearly all amino acids increased. 

The pattern of change in plasma concentrations of phospholipids (lysophophatidylcholines (LPC), phosphatidylcholines (PC), and sphingomyelins (SM)) exhibited a very similar pattern (decreased immediately post-race, followed by significant elevations during 72 h of recovery) (Figure 2, Table S1).

### 2.3. Differences between Performer Groups

Plasma metabolite concentrations, ratios, and shifts were compared between the three performer groups. A total of 144 metabolites or metabolite ratios were significantly different (*p* < 0.05) between performer groups (Table S2). In most instances, these differences were detected at T3 (33 metabolites with *p* < 0.05; see Figure S3A,B) and T4 (69 metabolites with *p* < 0.05). However, only 14 metabolites and metabolite ratios remained significant after multiple test correction, with ten at T3 and two at T4 with regard to all performance groups (Figure 3, Table S2). Two metabolites, acetyl-ornithine (Ac-Orn) and lysophosphatidylcholine 18:2 (lysoPC18:2), were found to be significantly different between LP and TP at time points T1 and T5, respectively. 

The most pronounced differences between performer groups were observed at T3, particularly between TP and LP (Figure S3A,B, Figure 4, Table S2). Most notably, of the 33 metabolites found to be different between performer groups at T3, 19 were acylcarnitines, which were significantly higher in LP (Figure S3A,B). Those acylcarnitines carrying the most abundant plasma fatty acids (palmitic acid, C16; stearic acid, C18; oleic acid, C18:1) remained significant after multiple test correction (Figure 3, Figure 4). Furthermore, at T3 the CPT1 ratio ((C18 + C16)/C0) was significantly different between the performer groups and was highest in LP (log2 fold changes (FC) = 1.44, *q =* 1.1 × 10^−3^) in comparison to AP (log2 FC = 1.37, *q =* 1.6 × 10^−6^) and TP (log2 FC = 0.86, *q =* 1.6 × 10^−3^). 

**Figure 5:**
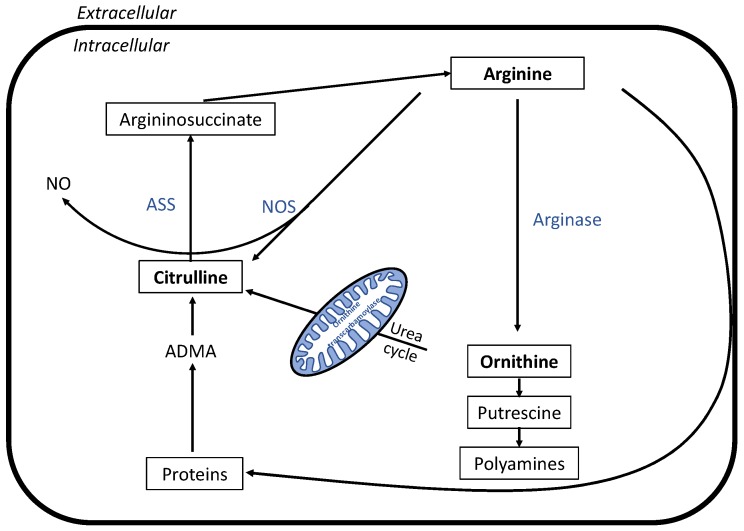
Relevant metabolic pathways involved in exercise-induced changes of arginine metabolism. Black fonts represent metabolites, blue font enzymes. ASS: argininosuccinate synthase, NOS: nitric oxide synthase, NO: nitric oxide, ADMA: asymmetric dimethylarginine.

Six metabolites and metabolite ratios, that were significantly different mainly between TP and AP/LP, were related to arginine metabolism pathways (Arg, Cit/Arg, Cit/Orn, Glu, Orn, Orn/Arg) (Figure 4, Figure 5). These metabolites and ratios were not significantly changed in the TP group between T1 and T3 (Figure 4, Table S1), in contrast to significant changes in the LP and AP groups. For instance, in the LP group we found Arg (FC = −2.0, *q* = 8.9 × 10^−5^), Orn/Arg (FC = 2.3, *q* = 8.9 × 10^−5^), Cit/Arg (FC = 1.4, *q* = 8.9 × 10^−5^), and Cit/Orn (FC = −0.9, *q* = 8.9 × 10^−5^) to be significantly altered between T1 and T3.

## 3. Discussion

This study used a targeted metabolomics approach to compare shifts in plasma concentrations of acylcarnitines (AC), amino acids (AA), phospholipids, sphingolipids, the sum of hexoses over a training period of five weeks and three days of recovery in 76 marathon runners divided into three groups based on fitness and race performance levels. The results support marked immediate post-race increases for plasma concentrations of AC and decreases in most AA and phospholipids, followed by a strong elevation in AA and phospholipids at 24 and 72 h post-race. Immediate post-race plasma concentrations of AC, the CPT1 ratio, and arginine-related metabolites were higher in the low compared to top performers. 

### 3.1. Metabolic Changes in Different Exercise Periods and Performance Groups

The final pathways of energy metabolism including β-oxidation, tricarboxylic acid cycle, and oxidative phosphorylation via the electron transport chain take place in the mitochondrion. Exercise stimulates mitochondrial growth [14]. Mitochondria adapt to the metabolic stress induced by specific exercise [15]. High-intensity interval training improves mitochondrial respiratory function [16] whereas endurance training increases the mitochondrial content [17]. The data from this study will be described within this context.

### 3.2. Acylcarnitines

In a previous laboratory-based study, acylcarnitines were identified as markers of increased fat utilization during 1–2 h of treadmill running [18]. Specifically, levels of octanoylcarnitine (C8), decanoylcarnitine (C10), and dodecanoylcarnitine (C12), and free fatty acids were elevated after exercise. Lehmann et al. interpreted these findings as an indication for high rates of fatty acid oxidation and low reliance on glycolysis [18].

Our data support a strong AC signature immediately after running a marathon, especially in the group of low performers compared to the other performance groups. There are two explanations for these findings. During prolonged and intensive running, lipolysis, proteolysis, glycogen depletion, and hepatic gluconeogenesis are enhanced in marathon runners to supply the body with sufficient energy [10]. More precisely, β-oxidation and the tricarboxylic acid cycle (TCA) are activated to deliver substrates for ATP production [12]. At lower exercise intensities (50%–60% VO_2_max), fat and carbohydrate oxidation both contribute to ATP generation. At higher exercise intensities (>60% VO_2_max), glycolysis is enhanced, with acidosis, a decrease in free Coenzyme A (CoA) and free carnitine, and accumulation of acetyl-CoA [19,20]. The data from our study showed log2-fold increases of 1.5 and 1.7 for the C2/C0 ratio, which might reflect activity of the mitochondrial enzyme carnitine acetyltransferase (CAT). CAT sequesters acetyl-CoA to free carnitine that serves as an acceptor for the transport of long-chain fatty acids via carnitine palmitoyltransferase (CPT1) into the mitochondrion. Consequently, the import of free fatty acids into the mitochondrion, where β-oxidation takes place, is impaired at higher exercise intensities due to an excess of acetyl-CoA [21]. In this context, fatty acid oxidation decreases, and acylcarnitine and free fatty acids accumulate [22].

The increase in acylcarnitines after the race, especially in LP, could also be related to glycogen depletion and an increased reliance on lipolysis for ATP production. Increased lipolysis leads to a higher concentration of fatty acids and subsequently to higher concentrations of acylcarnitines in the mitochondrion [18]. With the increase in lipolysis and β-oxidation, acetyl-CoA is diverted to supporting ketogenesis or the TCA-cycle, and plasma levels of acylcarnitines increase, as has been shown in individuals who are obese or with type II diabetes mellitus [23,24]. The higher post-race concentrations of plasma acylcarnitines in the LP compared to TP groups was more than likely related to a lower capacity to mobilize and oxidize lipids during prolonged and intense exercise, faster glycogen depletion rates, and longer race times. 

### 3.3. Amino Acids, Arginine Metabolism- and Urea Cycle Related-Metabolites, and Biogenic Amines

Ishikura et al. described significant post-exercise changes in plasma amino acid concentrations to support metabolic needs [25]. Amino acids decreased significantly immediately after the marathon race to support gluconeogenesis and ATP production through the TCA cycle in response to glycogen-depletion, as has been reported by others [25,26]. Plasma hexose was higher in the TP group after the race (T3), potentially indicating an optimized gluconeogenesis in trained athletes. These findings corroborate prior studies indicating a reduction in glycogenic amino acids immediately after prolonged exercise [10,27]. 

As proteins break down to amino acids that enter the TCA cycle as substrates, the liberated nitrogen is shifted to the urea cycle. Arginine is the central substrate of the urea cycle [28,29], and our data showed lower post-race levels, especially in the LP group. Figure 5 shows the involvement of ornithine and citrulline in the arginine metabolic pathway. Citrulline is a product of arginine after nitrogen oxide (NO) is generated, and post-race plasma levels were decreased especially in the LP group. This could have been due to limited arginine levels or enhanced NO production because of the long race duration in the LP group [30]. Additionally, arginine is a glucogenic amino acid that contributes to gluconeogenesis. Prolonged and strenuous exercise decreases asymmetric dimethylarginine (ADMA) [31], one of the substrates for citrulline synthesis. 

Post-race levels of ornithine were increased in the LP and AP groups in contrast to a decrease in the TP group. These group differences could be attributed to elevated muscle mitochondrial number and function in the TP group, and corresponding effects on the urea cycle and ornithine involvement [15,16,17]. Lactate inhibits urea synthesis during the transformation to arginine, and the faster runners could have accumulated more lactate, resulting in lower formation of urea and lower plasma ornithine levels [32].

The post-race increase in plasma glutamate could be related to exercise-induced muscle damage and subsequent leakage into the blood [33]. Other studies have reported elevated blood concentrations of glutamate in healthy untrained volunteers and endurance athletes after an incremental cycling test to exhaustion [34]. 

### 3.4. Sphingolipids and Phospholipids

The post-race decreases in plasma phosphatidylcholine and lysophosphatidylcholine levels indicate a higher transport rate of fatty acids to support the exercise-induced increase in lipolysis [10]. Newsom et al. observed an exercise-induced decrease of muscular phophatidylcholine content in athletes after exercise [35]. Lee et al. investigated the effect of treadmill exercise on muscular lipid profiles in rats including phospholipids, sphingolipids, ceramides, diacylglycerols (DAGs), and triacylglycerols (TAGs). They observed an exercise-induced decrease in most lipid levels [10]. 

### 3.5. Limitations of the Study

This study used a targeted metabolomics approach that provided quantitative data on selected AC, AA, hexoses, phospholipids, and sphingolipid metabolites. An untargeted metabolomics approach would have provided information on other types of metabolites, but without absolute quantification.

Furthermore, metabolite concentrations were measured in blood plasma, providing a glimpse into the exercise metabolism, whereas molecular methodologies with isotope-labeled tracer molecules could have provided profound insight into metabolite kinetics [36].

The runner groups included only males. We excluded females because this was a substudy of the Enzy-MaGIC trial, in which hormone-dependent parameters were also investigated. 

One major challenge in our study was the demographic differences between the performer classes. It is plausible that younger runners with lower body fat are better athletes than older runners with higher body fat, and this might have had an impact on our data analysis and interpretation. However, the fact that there was only one significantly different metabolite between performer classes at the time points before the race (T1 and T2) shows that the metabolite levels in the unchallenged runners were comparable between performer classes. We thus conclude that the differences at time points T3, T4, and T5 are mainly based on differences in training levels.

Participants were fasted for all blood collection samples except immediately after finishing the race, and the potential influence of carbohydrate intake during the race was not measured (although a standardized carbohydrate ingestion was recommended).

Group stratification was based on race performance and peak oxygen uptake, and the findings could have been influenced by the arbitrary cut-off values.

## 4. Materials and Methods 

### 4.1. Subjects and Procedures

This is a substudy of the Enzy-MagIC study, which was a randomized, double-blinded, placebo-controlled trial, as previously described [37]. Participants randomly received either Wobenzym^®^ plus (an enzyme-rutoside combination, MUCOS Pharma, Berlin, Germany) or a placebo [37,38]. The intervention had no significant effect on metabolite shifts induced by running the Munich Marathon, and this analysis proceeded as described in this paper. The study was approved by the ethics committee of the University Hospital Klinikum rechts der Isar, Munich, Germany (approval reference number 5820/13) and the Federal Institute for Drugs and Medical Devices, Germany (approval reference number 4039219). The study was in accordance with the Declaration of Helsinki. The trial was registered at ClinicalTrials.gov (NCT01916408).

#### 4.1.1. Recruitment of Subjects

A total of 166 healthy male athletes, aged between 20 and 65 years, who participated in the Munich Marathon 2013, were assessed for eligibility (Figure S1). Out of this panel, 162 met the inclusion criteria and were included in the study collective as described elsewhere [38]. One hundred forty of these 162 participants finished the marathon and completed the cardiopulmonary exercise testing beforehand. We were able to gather the complete data sets of all visits including blood samples from 127 runners.

#### 4.1.2. Separation of Subjects into Different Performance Groups

Maximum oxygen consumption (VO_2_max) was measured for all subjects during a cardiopulmonary exercise treadmill test to volitional exhaustion during their first visit (T1) (Pulsar 3P, h/p/cosmos sports and medical GmbH, Traunstein, Germany). VO_2_max was assessed by using a gas exchange device with breath-by-breath technology (Cortex MetaLyzer 3B, Leipzig, Germany). The exercise testing protocol was performed as follows: after a six-minute warm-up, an individually adjusted ramp protocol was used to exhaust subjects within 10 to 14 min. Complete exhaustion of participants during the test was defined as reaching an oxygen consumption (VO2) plateau despite an increase in the workload.

The participants (*n* = 127) were stratified into three groups based on aerobic fitness level and race performance. These groups included top (*n* = 20, top performers, TP), average (*n* = 87, average performers, AP), and low (*n* = 20, low performers, LP) performers based on endurance capacity (relative VO_2_max) and net marathon finishing time (Figure 1). Out of the 87 AP, 40 participants were randomly selected (to reduce costs). Thus, metabolomics analyses were applied to 80 athletes in total, with four participants removed (two from TP, two from LP) due to analysis issues. The quotient of the finishing time (cohort mean: 225.1 ± 43.1 min) and VO_2_max (cohort mean: 51.2 ± 9.4 mL × min^−1^ × kg^−1^) was used as an estimate of performance and fitness. 

#### 4.1.3. Blood Samples 

Blood samples for the metabolomics approach were collected five weeks (T1) and one week (T2) before the race and immediately (T3), 24 h (T4), and 72 h (T5) after the race. All blood samples were measured for the TP and LP groups, with T1, T3, and T4 measured for the AP group. All samples were collected in a fasting state except for the immediate post-race sample. Participants were supplied with up to two sodium-rich gels (PowerBar Gel Extra-Natrium: 0.5 g Na + /100g) per hour during the marathon, which contain 54 g of carbohydrates per hour of exercise. Blood samples at visits T1, T2, T4, and T5 were acquired in a fasted state. Immediately post-race (T3) blood samples were taken before post-race nutritional intake, nevertheless, participants cannot be considered as completely fasted after gel consumption during the race. Furthermore, participants were instructed not to substantially change their nutritional habits during the seven weeks of the Enzy-MagIC Study. 

#### 4.1.4. Sample Handling and Metabolite Quantification

T1 and T2 blood samples were aliquoted immediately after blood withdrawal and stored at −80 °C at the Genome Analysis Center at the Helmholtz Zentrum Muenchen until sample analysis. T3, T4, and T5 blood samples were stored at 4 °C after collection, aliquoted within one hour, and then frozen and stored at −80 °C. To ensure reproducibility, sample handling was performed with a Hamilton Microlab STARTM robot (Hamilton Bonaduz AG, Bonaduz, Switzerland) and an Ultravap nitrogen evaporator (Porvair Sciences, Leatherhead, UK). Mass spectrometric analyses were done with a Sciex API 4000 triple quadrupole system (Sciex Deutschland GmbH, Darmstadt, Germany) equipped with a 1200 Series HPLC (Agilent Technologies Deutschland GmbH, Böblingen, Germany) and an HTC PAL auto sampler (CTC Analytics, Zwingen, Switzerland) controlled by the software Analyst 1.6.2. 

Targeted metabolomics analyses were based on LC-ESI-MS/MS (liquid chromatography-electrospray ionisation-triple quadrupol mass spectrometry) and FIA (flow injection)-ESI-MS/MS measurements using the Biocrates Absolute*IDQ*^TM^ p180 Kit (BIOCRATES Life Sciences AG, Innsbruck, Austria). The assay is in conformance with the EMEA-Guideline “Guideline on bioanalytical method validation (July 21st 2011)” [39] and allows for the quantification of 188 metabolites including free carnitine, 39 acylcarnitines, 21 amino acids, 21 biogenic amines, 1 sum of hexoses, 90 glycerophospholipids, and 15 sphingolipids. Analytical specifications including limit of detection (LOD), lower limit of quantification (LLOQ), upper limit of quantification (ULOQ), linearity, reproducibility, and metabolite stability are described in the Biocrates manual AS-P180. The assay procedures as well as the metabolite nomenclature have been described in detail previously [40,41] and are documented in the Biocrates manual UM-P180. Metabolite data evaluation and quality assessment were performed with the software MultiQuant 3.0.2 (Sciex) and the Biocrates Met*IDQ*™ software package. Metabolite concentrations were calculated by comparing peak area ratios between analytes and the respective internal standards and are reported in µM. In addition to the investigated samples, five aliquots of an external pooled reference plasma were analyzed in each batch and used for data normalization.

### 4.2. Statistical Methods

R version 3.6.1 was used for data processing and statistical calculations. To ensure high data quality, metabolomics data were pre-processed as follows: First, metabolites with more than 40% of missing data points were removed from the data set (15 metabolites). In a second step, metabolites with a coefficient of variation >25% were removed (24 metabolites). After data cleaning, 193 metabolites and metabolite ratios remained in the data set.

Missing values (3.9%) were imputed by first dividing the minimum observed value of the respective metabolite by the square root of 2. The resulting value was then multiplied with a random factor between 0.75 and 1.25.

Fold changes between time points within performer classes were computed by using log2 transformed metabolite concentrations. Two-sided Wilcoxon rank sum tests were used for statistical analysis of comparisons between time points (paired test) and performer classes (unpaired test), respectively. The *p* values were corrected using the Benjamini–Hochberg method [42]. Comparison of phenotypic data was performed using a *t*-test after testing for normal distribution. Level of significance was set at 0.05 for all statistical tests.

## 5. Conclusions

This study demonstrated that participation in the Munich Marathon was associated with an extensive, prolonged perturbation in plasma concentrations of AC, AA, hexoses, phospholipids, and sphingolipid metabolites in 76 male runners. Plasma AC concentrations were significantly elevated post-exercise, and gradually decreased to near pre-race levels during 72 h of recovery. In contrast, plasma AA and phospholipid metabolite concentrations were generally decreased immediately post-race, followed by sharp increases during 72 h of recovery. These metabolite shifts were compared between three groups stratified by aerobic capacity and race performance. This analysis revealed that post-race plasma concentrations of AC and arginine-related metabolites were higher in the low compared to top performers, reflecting differences in the capacity to oxidize lipids for energy production during prolonged and intensive running.

## Figures and Tables

**Table 1:** Baseline characteristics of the study participants.

Performer	Low [*n* = 20]	Average [*n* = 40]	Top [*n* = 20]	*p* Value			Total [*n* = 80]
**Anthropometry**				Top vs. Low	Top vs. Avg	Avg vs. Low	
Age [year]	50.5 ± 7.9	44.0 ± 9.2	32.8 ± 7.9	5.99 × 10^−08^	1.27 × 10^−05^	1.14 × 10^−02^	42.8 ± 10.8
BMI [kg/m^2^]	25.3 ± 2.9	23.4 ± 2.3	21.9 ± 1.3	2.17 × 10^−05^	1.12 × 10^−02^	1.16 × 10^−02^	23.5 ± 2.5
Body fat Stander	17.6 ± 3.7	14.0 ± 4.1	8.3 ± 1.7	1.90 × 10^−12^	8.50 × 10^−08^	1.68 × 10^−03^	13.5 ± 4.9
**Performance**							
Marathon time	276.6 ± 29.6	224.6 ± 22.7	174.5 ± 17.0	5.96 × 0^−16^	7.65 × 10^−13^	1.06 × 10^−07^	225.1 ± 43.1
VO_2_max, absolute [l/min]	3.5 ± 0.7	3.7 ± 0.4	4.3 ±0.4	8.52 × 10^−06^	1.43 × 10^−07^	3.35 × 10^−01^	3.8 ± 0.5
VO_2_max, relative [mL × min^−1^ × kg^−1^]	41.8 ± 5.5	50.0 ± 5.7	63.3 ± 5.2	6.28 × 10^−16^	2.60 × 10^−11^	1.20 × 10^−06^	51.2 ± 9.4
**Training history**							
Training kilometers 2013 [km/year]	831.5 ± 471.9	1713.3 ± 1082.5	2420.5 ± 1128.8	1.91 × 10^−05^	4.13 × 10^−02^	4.19 × 10^−03^	1694.8 ± 1125.8

## Supplementary Materials

The following are available online at https://www.mdpi.com/2218-1989/10/3/87/s1, Figure S1: Flow diagram of included study participants, Figure S2: Performer class specific log2-fold changes referenced to time point 1. * *p* < 0.05, ** *p* < 0.01, *** *p* < 0.001, Quantification of 188 metabolites including acylcarnitines, amino acids, biogenic amines, hexoses, phospholipids (lysophosphatidylcholines, phosphatidylcholines), sphingolipids (sphingomyelins), and metabolite sums and ratios. Groups included top (*n* = 20, top performers, TP), average (*n* = 40, average performers, AP), and low (*n* = 20, low performers, LP) performers based on endurance capacity (relative VO_2_max) and net marathon finishing time. Blood samples were collected at time points T1 (training phase), T2 (tapering phase), T3 (immediately after the race), T4 (24 h post-race), and T5 (72 h post-race). All blood samples were measured for the TP and LP groups, with T2, T3, and T4 measured for the AP group, Figure S3: Metabolite concentrations with regard to the different cohorts. Thirty-three metabolites with significant differences (*p* < 0.05) between performer classes. Metabolites/ratios with q-values < 0.05 are denoted as †. 19 metabolites/ratios belong to acylcarnitines, 8 can be attributed to the arginine metabolism. * *p* < 0.05, ** *p* < 0.01, *** *p* < 0.001, NS. Non-significant. (A). Significant differences regarding absolute values at T3 (immediately after the race). Asterisks on the left side denote a significant difference between low and average performers, asterisks in the middle denote a significant difference between low and top performers, and asterisks on the right side denote a significant difference between average and top performers. (B). Significant differences regarding changes from T1 to T3. Significance levels refer to the differences from T1 to T3 within each performer group, Table S1: Metabolites with significantly different (*p* < 0.05) log2-fold changes of T1 normalized time points within the performer classes, Table S2: Metabolites with significantly different (*p* < 0.05) log2-fold changes of T1 normalized time points between the performer classes.
