# Supplementary material for: Metabolite Shifts Induced by Marathon Race Competition Differ between Athletes Based on Level of Fitness and Performance: A Substudy of the Enzy-MagIC Study
Source: Metabolites. 2020 Mar 1;10(3):87. doi: 10.3390/metabo10030087 (PMC7143325; doi:10.3390/metabo10030087)
Supplement: Supplementary file 1 [file metabolites-10-00087-s001.zip › Supplementary/Figure S3A.pdf]

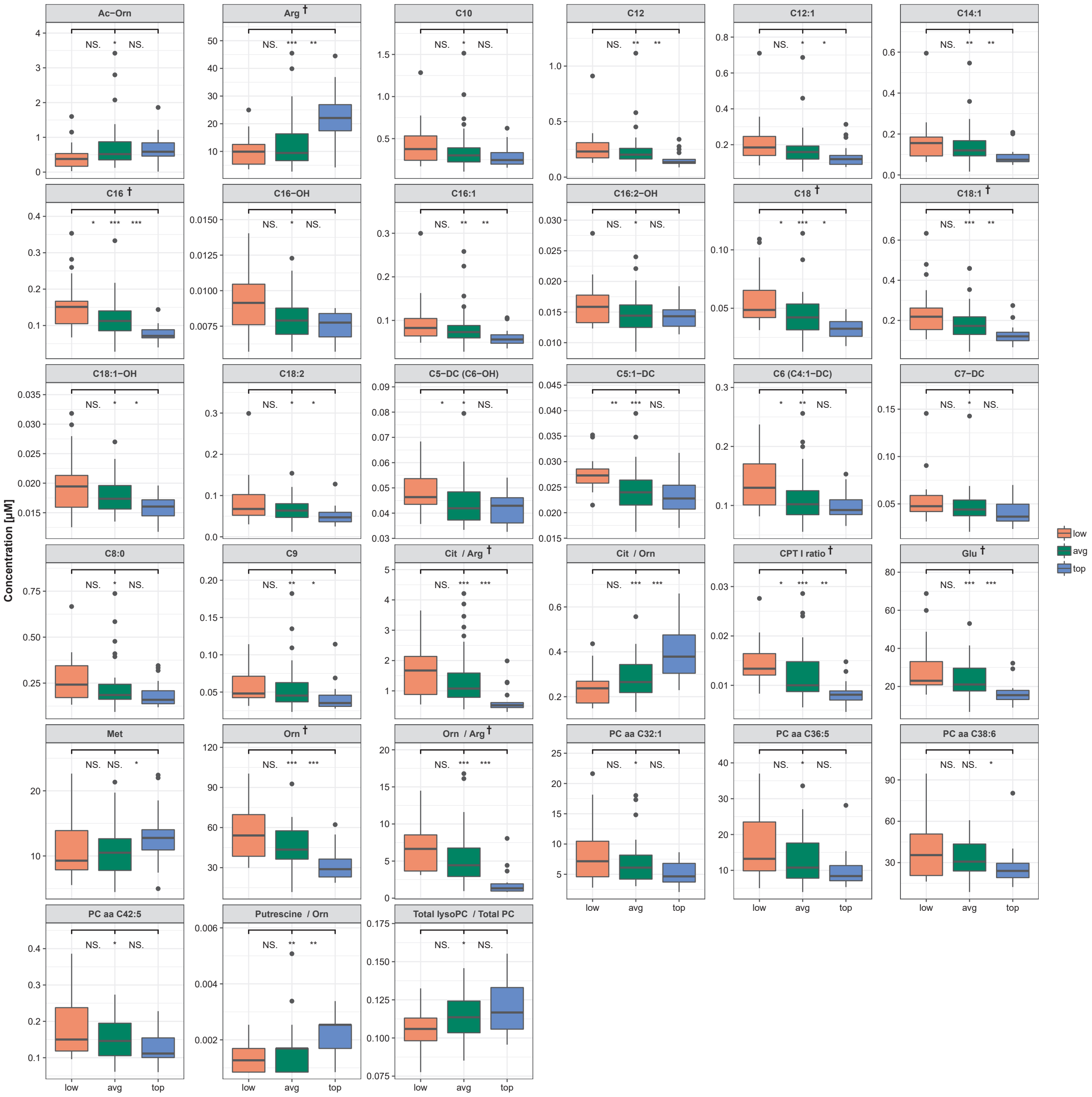

Figure S3A: Metabolite concentrations with regard to the different cohorts. Thirty-three metabolites with significant differences ($p < 0.05$) between performer classes. Metabolites/ratios with q-values < 0.05 are denoted as †. 19 metabolites/ratios belong to acylcarnitines, 8 can be attributed to the arginine metabolism. * $p < 0.05$, ** $p < 0.01$, *** $p < 0.001$, NS. Non-significant. Significant differences regarding absolute values at T3 (immediately after the race). Asterisks on the left side denote a significant difference between low and average performers, asterisks in the middle denote a significant difference between low and top performers, and asterisks on the right side denote a significant difference between average and top performers.
