# Supplementary material for: Metabolite Shifts Induced by Marathon Race Competition Differ between Athletes Based on Level of Fitness and Performance: A Substudy of the Enzy-MagIC Study
Source: Metabolites. 2020 Mar 1;10(3):87. doi: 10.3390/metabo10030087 (PMC7143325; doi:10.3390/metabo10030087)
Supplement: Supplementary file 1 [file metabolites-10-00087-s001.zip › Supplementary/Figure S3B.pdf]

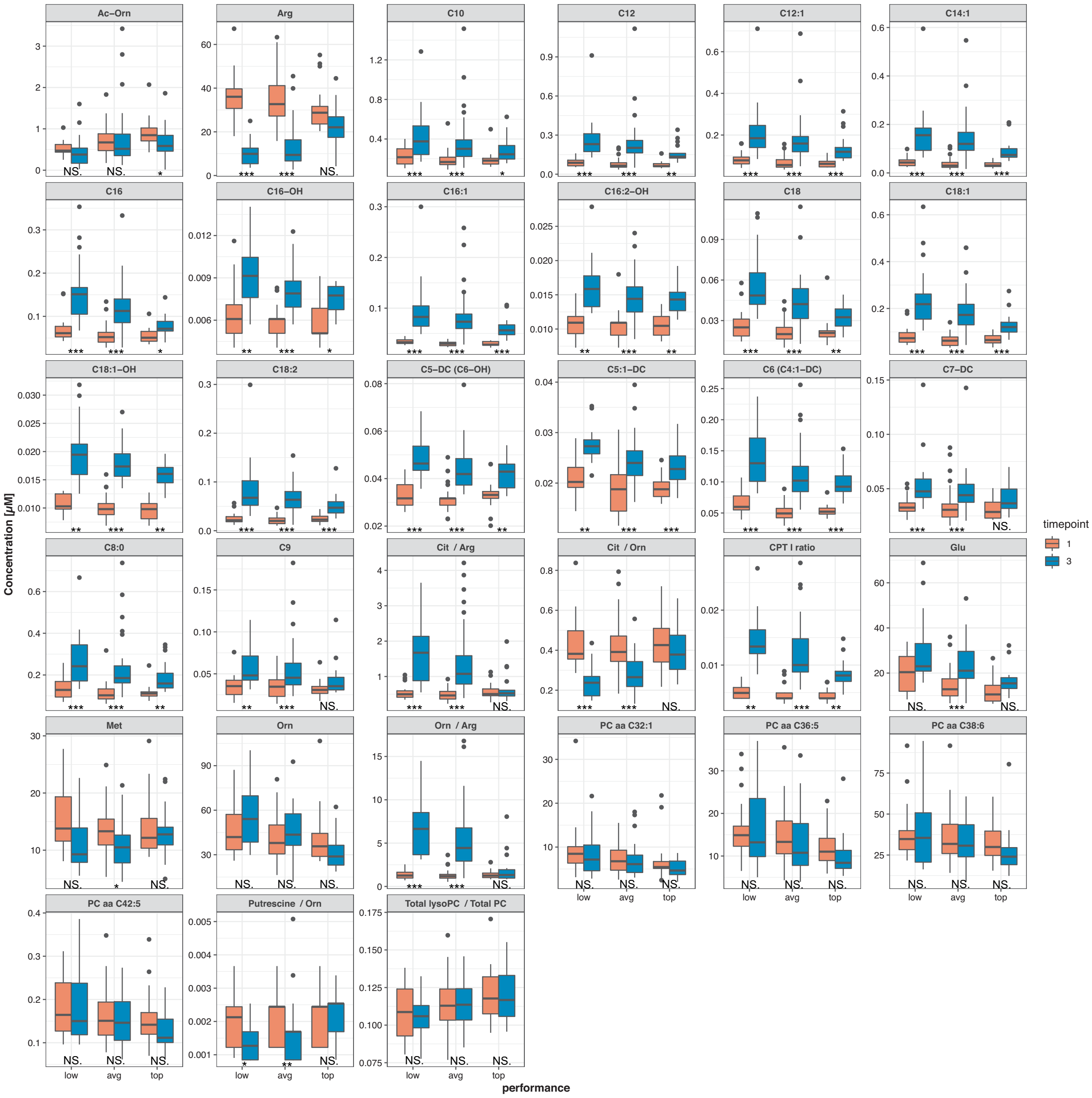

Figure S3B. Metabolite concentrations with regard to the different cohorts. Thirty-three metabolites with significant differences ($p < 0.05$) between performer classes. Metabolites/ratios with q-values < 0.05 are denoted as †. 19 metabolites/ratios belong to acylcarnitines, 8 can be attributed to the arginine metabolism. * $p < 0.05$, ** $p < 0.01$, *** $p < 0.001$, NS. Non-significant. (B). Significant differences regarding changes from T1 to T3. Significance levels refer to the differences from T1 to T3 within each performer group, Table S1. Metabolites with significantly different ($p < 0.05$) log2-fold changes of T1 normalized time points within the performer classes, Table S2: Metabolites with significantly different ($p < 0.05$) log2-fold changes of T1 normalized time points between the performer classes.
