# Supplementary material for: Metabolite Shifts Induced by Marathon Race Competition Differ between Athletes Based on Level of Fitness and Performance: A Substudy of the Enzy-MagIC Study
Source: Metabolites. 2020 Mar 1;10(3):87. doi: 10.3390/metabo10030087 (PMC7143325; doi:10.3390/metabo10030087)
Supplement: Supplementary file 1 [file metabolites-10-00087-s001.zip › Supplementary/Figure S2.pdf]

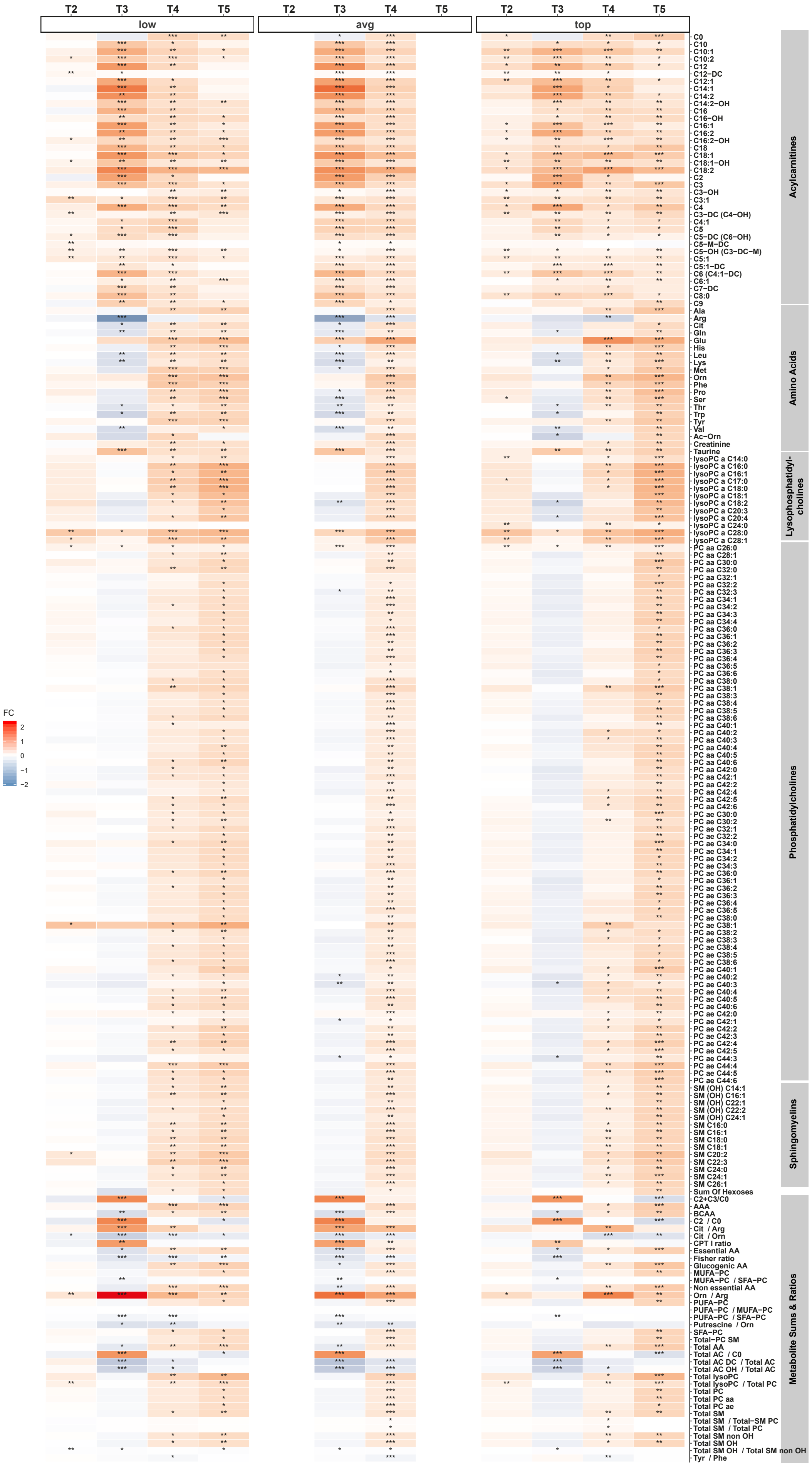

T2
T3
T4
T5
low
avg
top
FC
2
1
0
−1
−2
Acylcarnitines
Amino Acids
Lysophosphatidyl-cholines
Phosphatidylcholines
Sphingomyelins
Metabolite Sums & Ratios
C0
C10
C10:1
C10:2
C12
C12−DC
C12:1
C14:1
C14:2
C14:2−OH
C16
C16−OH
C16:1
C16:2
C16:2−OH
C18
C18:1
C18:1−OH
C18:2
C2
C3
C3−OH
C3:1
C4
C3−DC (C4−OH)
C4:1
C5
C5−DC (C6−OH)
C5−M−DC
C5−OH (C3−DC−M)
C5:1
C5:1−DC
C6 (C4:1−DC)
C6:1
C7−DC
C8:0
C9
Ala
Arg
Cit
Gln
Glu
His
Leu
Lys
Met
Orn
Phe
Pro
Ser
Thr
Trp
Tyr
Val
Ac−Orn
Creatinine
Taurine
lysoPC a C14:0
lysoPC a C16:0
lysoPC a C16:1
lysoPC a C17:0
lysoPC a C18:0
lysoPC a C18:1
lysoPC a C18:2
lysoPC a C20:3
lysoPC a C20:4
lysoPC a C24:0
lysoPC a C28:0
lysoPC a C28:1
PC aa C26:0
PC aa C28:1
PC aa C30:0
PC aa C32:0
PC aa C32:1
PC aa C32:2
PC aa C32:3
PC aa C34:1
PC aa C34:2
PC aa C34:3
PC aa C34:4
PC aa C36:0
PC aa C36:1
PC aa C36:2
PC aa C36:3
PC aa C36:4
PC aa C36:5
PC aa C36:6
PC aa C38:0
PC aa C38:1
PC aa C38:3
PC aa C38:4
PC aa C38:5
PC aa C38:6
PC aa C40:1
PC aa C40:2
PC aa C40:3
PC aa C40:4
PC aa C40:5
PC aa C40:6
PC aa C42:0
PC aa C42:1
PC aa C42:2
PC aa C42:4
PC aa C42:5
PC aa C42:6
PC ae C30:0
PC ae C30:2
PC ae C32:1
PC ae C32:2
PC ae C34:0
PC ae C34:1
PC ae C34:2
PC ae C34:3
PC ae C36:0
PC ae C36:1
PC ae C36:2
PC ae C36:3
PC ae C36:4
PC ae C36:5
PC ae C38:0
PC ae C38:1
PC ae C38:2
PC ae C38:3
PC ae C38:4
PC ae C38:5
PC ae C38:6
PC ae C40:1
PC ae C40:2
PC ae C40:3
PC ae C40:4
PC ae C40:5
PC ae C40:6
PC ae C42:0
PC ae C42:1
PC ae C42:2
PC ae C42:3
PC ae C42:4
PC ae C42:5
PC ae C44:3
PC ae C44:4
PC ae C44:5
PC ae C44:6
SM (OH) C14:1
SM (OH) C16:1
SM (OH) C22:1
SM (OH) C22:2
SM (OH) C24:1
SM C16:0
SM C16:1
SM C18:0
SM C18:1
SM C20:2
SM C22:3
SM C24:0
SM C24:1
SM C26:1
Sum Of Hexoses
C2+C3/C0
AAA
BCAA
C2 / C0
Cit / Arg
Cit / Orn
CPT I ratio
Essential AA
Fisher ratio
Glucogenic AA
MUFA−PC
MUFA−PC / SFA−PC
Non essential AA
Orn / Arg
PUFA−PC
PUFA−PC / MUFA−PC
PUFA−PC / SFA−PC
Putrescine / Orn
SFA−PC
Total−PC SM
Total AA
Total AC / C0
Total AC DC / Total AC
Total AC OH / Total AC
Total lysoPC
Total lysoPC / Total PC
Total PC
Total PC aa
Total PC ae
Total SM
Total SM / Total−SM PC
Total SM / Total PC
Total SM non OH
Total SM OH
Total SM OH / Total SM non OH
Tyr / Phe

Figure S2: Performer class specific log2-fold changes referenced to time point 1. * $p < 0.05$, ** $p < 0.01$, *** $p < 0.001$, Quantification of 188 metabolites including acylcarnitines, amino acids, biogenic amines, hexoses, phospholipids (lysophosphatidylcholines, phosphatidylcholines), sphingolipids (sphingomyelins), and metabolite sums and ratios. Groups included top (n = 20, top performers, TP), average (n = 40, average performers, AP), and low (n = 20, low performers, LP) performers based on endurance capacity (relative VO2max) and net marathon finishing time. Blood samples were collected at time points T1 (training phase), T2 (tapering phase), T3 (immediately after the race), T4 (24 h post-race), and T5 (72 h post-race). All blood samples were measured for the TP and LP groups, with T1, T3, and T4 measured for the AP group.
