# Supplementary figures and images for: Metabolite Shifts Induced by Marathon Race Competition Differ between Athletes Based on Level of Fitness and Performance: A Substudy of the Enzy-MagIC Study
Source: Metabolites. 2020 Mar 1;10(3):87. doi: 10.3390/metabo10030087 (PMC7143325; doi:10.3390/metabo10030087)

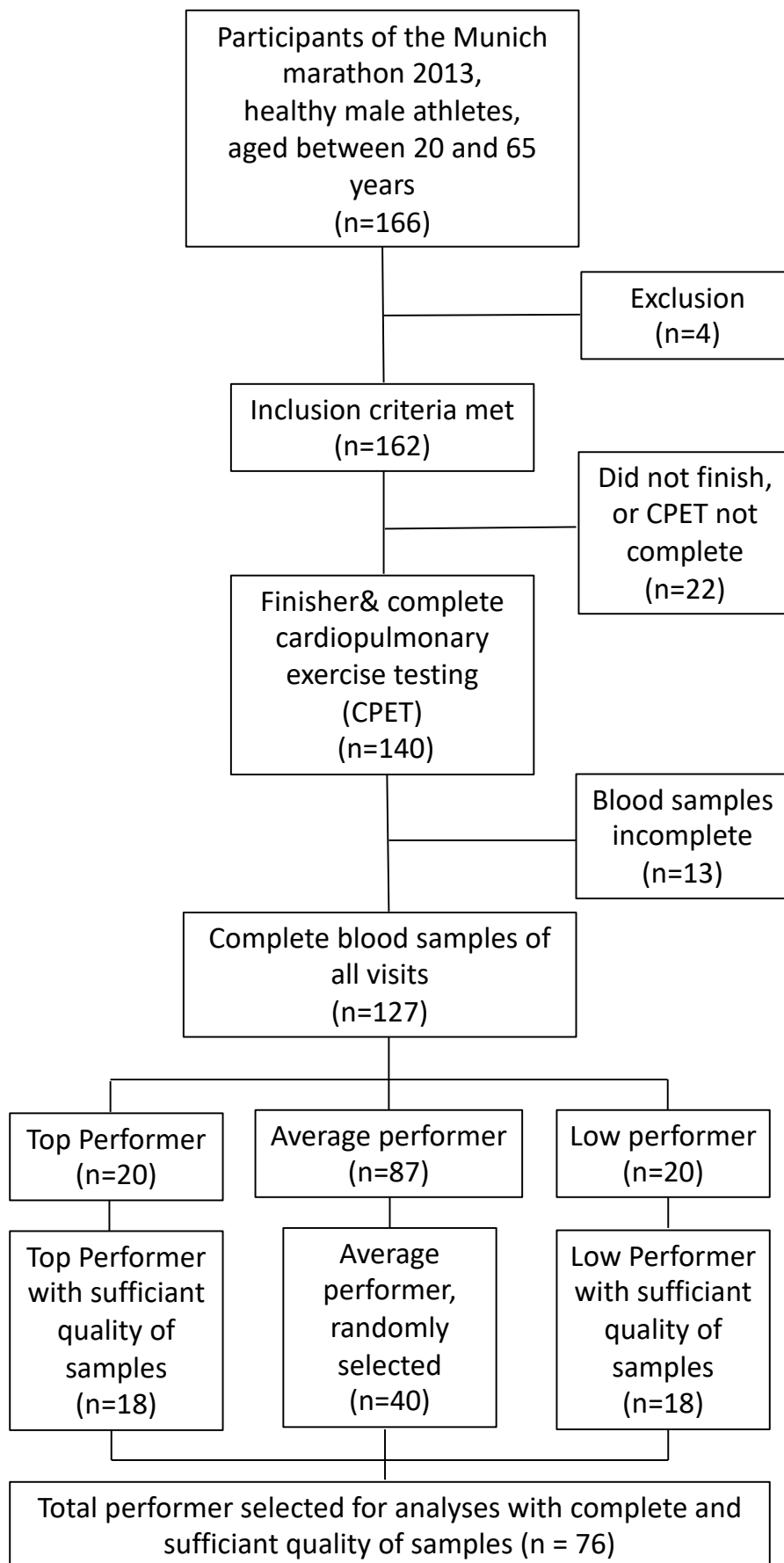

Figure S1: Flow diagram of included study participants

Supplement: Supplementary file 1 [file metabolites-10-00087-s001.zip › Supplementary/Figure S1.pdf]
